# Osteopathy

**Published:** 1910-10-08

**Authors:** George Garrard


*To the Editor of* The Hospital.

### OSTEOPATHY.

*To the Editor of* The Hospital.

Sir,—I am pleased to see that the subject of bone-setting, or mechano-therapy, is being discussed in your paper. As a surgeon may I be allowed to state my experience of Mr. H. A. Barker's methods? Some time ago I had a patient suffering from a painful and obscure affection of the ankle, which was causing her great pain and suffering, making walking almost impossible, and which for a long time refused to improve, though orthodox remedies were perseveringly tried. I advised her to see a famous London surgeon. She saw two at different times. She also went to Berlin and saw an eminent surgeon there. Their advice and treatment resulted in no improvement whatever.

When she returned she told me she would like to see Mr. H. A. Barker, as he had cured a friend of hers. I agreed, and she did so. At the end of a few weeks she made a complete recovery, relief being afforded almost immediately. My own son was at this time suffering from an ankle injury which also refused to yield to treatment by three surgeons at different times. It prevented him from indulging in any kind of sport at his University. Having already had experience of Mr. Barker's methods, I took my son to him and witnessed the treatment. The patient was put under gas, a few dexterous and determined manipulations of the joint were effected, and the patient was immediately all right. His words as he left the house were, "I've never been able to walk so well before." He has been quite well ever since, and now plays football and other games without feeling anything of the old trouble. I join Dr. Bryce heartily in pleading for the "admission of this scientific mechano-therapy or bone-setting amongst the recognised methods of treatment."

Yours, etc.,
George Garrard, M.R.C.S.Eng., L.R.C.P Lond.

